# Dr. John Homans

**Published:** 1903-03

**Authors:** 


Dr. John Homans, of Boston, died at his home in that city, February 7, 1903, aged 65 years. He graduated from Harvard College in 1858 and took his doctorate from Harvard Medical School in 1862. He was commissioned an assistant surgeon in the United States navy in 1862, and later in the same year he obtained the same rank in the army and rose to the rank of surgeon-in-chief of the first division of the 19th army corps, ultimately becoming medical inspector on the staff of General Sheridan.

Dr. Homans achieved fame as a surgeon in civil life, serving for many years on the staff of the Massachusetts Hospital, and as a teacher in Harvard Medical School. He was a member of the American Surgical Association and a Companion of the Military Order of the Loyal Legion of the United States.

